# Critical Roles of Circular RNA in Tumor Metastasis via Acting as a Sponge of miRNA/isomiR

**DOI:** 10.3390/ijms23137024

**Published:** 2022-06-24

**Authors:** Li Guo, Lin Jia, Lulu Luo, Xinru Xu, Yangyang Xiang, Yujie Ren, Dekang Ren, Lulu Shen, Tingming Liang

**Affiliations:** 1 Smart Health Big Data Analysis and Location Services Engineering Laboratory of Jiangsu Province, Department of Bioinformatics, School of Geographic and Biologic Information, Nanjing University of Posts and Telecommunications, Nanjing 210023, China; lguo@njupt.edu.cn (L.G.); 1221014238@njupt.edu.cn (Y.X.); 1021173610@njupt.edu.cn (Y.R.); 1021173609@njupt.edu.cn (D.R.); 2 Jiangsu Key Laboratory for Molecular and Medical Biotechnology, School of Life Science, Nanjing Normal University, Nanjing 210023, China; 201202100@njnu.edu.cn (L.J.); 211202099@njnu.edu.cn (L.L.); 211202068@njnu.edu.cn (X.X.); 191202064@stu.njnu.edu.cn (L.S.)

**Keywords:** circular RNA (circRNA), microRNA (miRNA), isomiR, cancer metastasis, competing endogenous RNA (ceRNA) network

## Abstract

Circular RNAs (circRNAs), a class of new endogenous non-coding RNAs (ncRNAs), are closely related to the carcinogenic process and play a critical role in tumor metastasis. CircRNAs can lay the foundation for tumor metastasis via promoting tumor angiogenesis, make tumor cells gain the ability of migration and invasion by regulating epithelial-mesenchymal transition (EMT), interact with immune cells, cytokines, chemokines, and other non-cellular components in the tumor microenvironment, damage the normal immune function or escape the immunosuppressive network, and further promote cell survival and metastasis. Herein, based on the characteristics and biological functions of circRNA, we elaborated on the effect of circRNA via circRNA-associated competing endogenous RNA (ceRNA) network by acting as miRNA/isomiR sponges on tumor angiogenesis, cancer cell migration and invasion, and interaction with the tumor microenvironment (TME), then explored the potential interactions across different RNAs, and finally discussed the potential clinical value and application as a promising biomarker. These results provide a theoretical basis for the further application of metastasis-related circRNAs in cancer treatment. In summary, we briefly summarize the diverse roles of a circRNA-associated ceRNA network in cancer metastasis and the potential clinical application, especially the interaction of circRNA and miRNA/isomiR, which may complicate the RNA regulatory network and which will contribute to a novel insight into circRNA in the future.

## 1. Background

**Figure 1:**
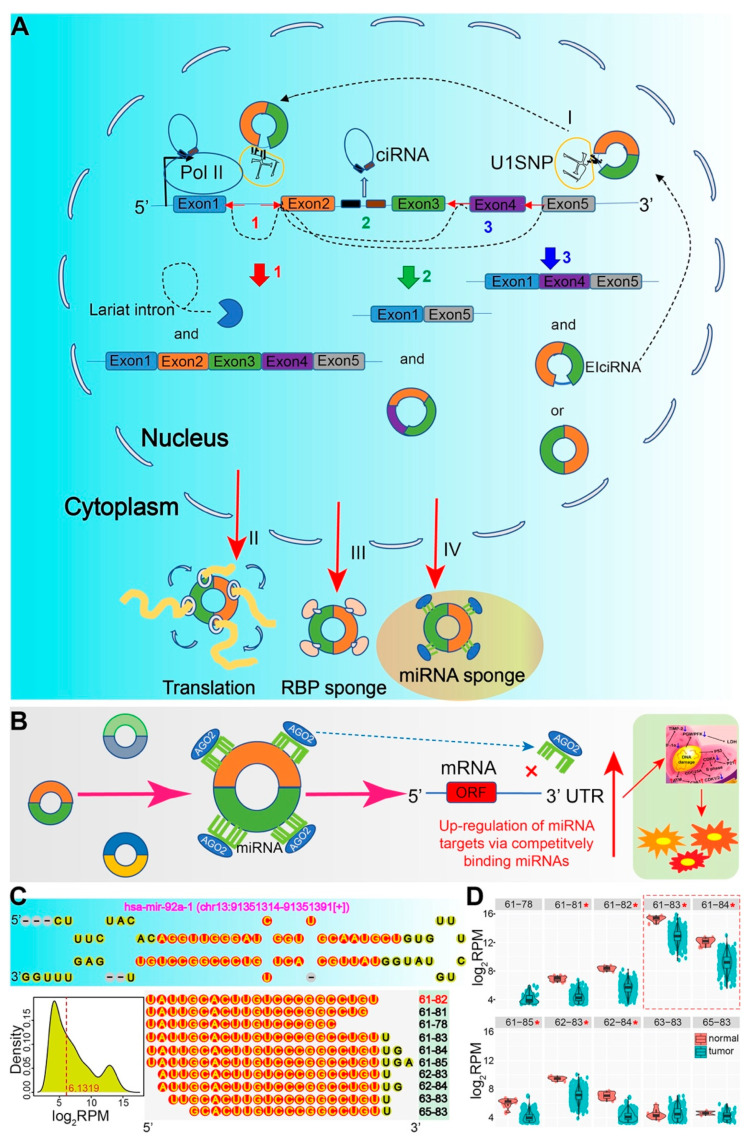
CircRNA biogenesis and multiple isomiRs in miRNA. (**A**): There are three types of circular RNAs: intronic circRNAs (ciRNA), exonic circRNAs with introns (EIciRNA), and exonic circRNA (ecircRNA). 1–3 indicates that alternative splicing of the same gene can produce three circRNAs; I-IV show that circRNA can regulate the expression of parent gene, acting as a miRNA sponge, interacting with RNA binding protein (RBP), and translating polypeptide, respectively; (**B**) CircRNA can bind to miRNA through miRNA response element (MRE), which further up-regulates the target gene regulated by miRNA. The dysregulation of target mRNAs may disturb relevant biological processes that can further contribute to cancer pathophysiology and developmental process; (**C**) Take hsa-mir-92a-1 as an example to demonstrate multiple isomiRs in miRNA locus due to alternative and imprecise cleavage of Drosha and Dicer, 3′ addition events, etc. The detailed expression data are derived from the colon adenocarcinoma cohort in TCGA (TCGA-COAD). The pre-miRNA stem-loop structure is presented, and the total expression distribution of multiple isomiRs in the miR-92a-3p locus is presented for all relevant isomiRs if reads per million mapped reads (RPM) >= 10. Ten dominantly expressed isomiR types are further screened if the isomiR type is detected in more than 40 individuals and has higher expression levels (the detailed locations on chromosomes are used to name the isomiR type). The highlighted red locations, 61–82 (chr13:91351361–91351382[+]), are the canonical miRNA sequence in the miRbase database [16,17]; (**D**) The detailed expression distributions for the ten isomiR types between normal and tumor samples. Except for 3 isomiR types, others show a significant expression difference in tumor samples than that in normal samples (significant down-regulated, padj < 0.05). Of these, the isomiRs of 61–83 and 61–84 are unexpectedly dominantly expressed, despite the fact that they are not canonical miRNA sequences. * indicates padj < 0.05 based on DESeq2 algorithm [18].

In recent years, non-coding RNAs (ncRNAs) have been widely concerned because of their regulatory roles in multiple biological processes, mainly including microRNAs (miRNAs), long non-coding RNAs (lncRNAs), and circular RNAs (circRNAs). CircRNA, a new type of endogenous ncRNA, is closely related to the development of diseases and has become a research hotspot. CircRNAs are mostly produced by the cyclization of exons, however, some circRNAs can also originate from introns, antisense RNA, and 5′ or 3′ untranslated and intergenic genomic regions (Figure 1A). CircRNAs can form covalent closed-loop structures through reverse splicing. Unlike linear RNAs with a 5′ cap and 3′ tail structure (mRNAs, miRNAs, and lncRNAs, etc.), circRNAs lack free 5′ and 3′ ends, and are highly resistant to exonuclease RNase R. Thus, circRNAs are more stable than other linear RNAs. It is found that the average half-life of circRNAs in plasma exceeds 48 h, far exceeding the average of 10 h of mRNAs [1]. CircRNAs are expressed in a tissue-specific and developmental stage-specific manner, and the tissue specificity is related to the occurrence and progress of diverse human diseases, mainly including esophageal cancer, gastric cancer, liver cancer, colorectal cancer, breast cancer, cardiovascular diseases, and nervous system diseases [2]. CircRNAs can coordinate gene expression and produce polypeptides by acting as miRNA sponges (Figure 1B), interact with RNA binding protein (RBP), regulate transcription, and then participate in many pathological processes, especially the metastasis of circRNAs in cancer. CircRNA plays a critical role in the human carcinogenesis process via contributing to multiple biological processes and pathways, especially in cancer metastasis, which is a sign of a malignant tumor and the main cause of death in cancer patients. It is worth noting that the ability of cancer cells to invade and metastasize to other tissues and organs is the deadliest sign of malignant tumors and the most life-threatening factor for cancer patients. More than 90% of cancer-related deaths are caused by metastatic diseases rather than corresponding primary tumors [3].

In recent years, a theory of competitive endogenous RNAs (ceRNAs) was firstly proposed in 2001 [4], suggesting that lncRNAs, pseudogene RNAs, and circular RNAs contain a common site for binding to miRNAs. Thus, these RNAs can sequester and inactivate miRNAs via acting as miRNA sponges, which further regulates the expression of target mRNAs of miRNAs. The competitive binding of different RNAs and miRNAs via bait or sponge can form a ceRNA network of mutual regulation that may be vital for cells. In relevant circRNA studies, the interactions between circRNAs and miRNAs are one of the major methods that play a role in a regulatory network to further regulate target mRNAs. More importantly, miRNA is not a single miRNA sequence, but a series of isomiRs with sequence and expression diversities mainly caused by alternative and imprecise cleavage of Drosha and Dicer, and 3′ addition events in miRNA biogenesis [5,6,7,8] (Figure 1C,D). Multiple isomiRs complicate miRNAome and coding-non-coding RNA regulatory network, especially some isomiRs may perturb the RNA interaction network due to changed 5′ ends and seed sequences [9,10,11,12,13,14,15]. In order to systematically present the associations of circRNAs and cancers to discuss the potential roles of circRNAs in cancer pathophysiology and developmental process, we briefly summarize the latest circRNA-associated ceRNA network to understand cross-talks among different RNAs and their potential roles in cancer metastasis.

## 2. CircRNA Plays a Role in Tumorigenesis and Angiogenesis via Sponging miRNAs

The continuous proliferation of cancer cells gradually increases the tumor volume. When the tumor tissue expands more than 1–2 mm, the tumor core cannot get enough oxygen and nutrients. The tumor growth is then blocked, which usually leads to the growth of new blood vessels. Thus, the availability of nutrients and oxygen may be restored, which provides a way to detach from the original lesion and transfer to a distant place [19,20]. Angiogenesis is a critical step in tumor invasion and metastasis and plays a vital role in tumor progression. It has been found that circRNA can regulate tumor angiogenesis and promote cancer metastasis by targeting VEGF or other molecules related to angiogenesis. For example, overexpression of circFndc3b can increase the expression of VEGF-A through interaction with FUS in endothelial cells, enhance angiogenesis activity, reduce the apoptosis of myocardial cells and endothelial cells, and thus regulate the cardiac repair after myocardial infarction [21]. CircSMARCA5 acts as the sponge for SRSF1 in glioblastoma multiforme (GBM), regulates the splicing of *VEGFA*, and affects angiogenesis [22]. Circ3823 contributes to the growth, metastasis, and angiogenesis of colorectal cancer via involvement in miR-30c-5p/TCF7 axis [23]. EBV-encoded circular RNA LMP2A (EBV-circLMP2A) is expressed in EBV-associated gastric cancer (EBVaGC) and is associated with distant metastasis and poor prognosis. Knocking down EBV-circLMP2A can inhibit the formation and migration of human umbilical vein endothelial cells (HUVEC), reduce the expression of VEGFA and HIF1α in cancer cells under hypoxia, and further promote angiogenesis [24]. In addition, it was found that circFOXP1 promotes angiogenesis by regulating the miRNA-127-5p/CDKN2AIP signaling pathway in osteosarcoma [25]. Exosome circRNA can also regulate angiogenesis, such as circRNA-100338 promotes metastasis of hepatocellular carcinoma by enhancing invasiveness and angiogenesis [26].

**Figure 2:**
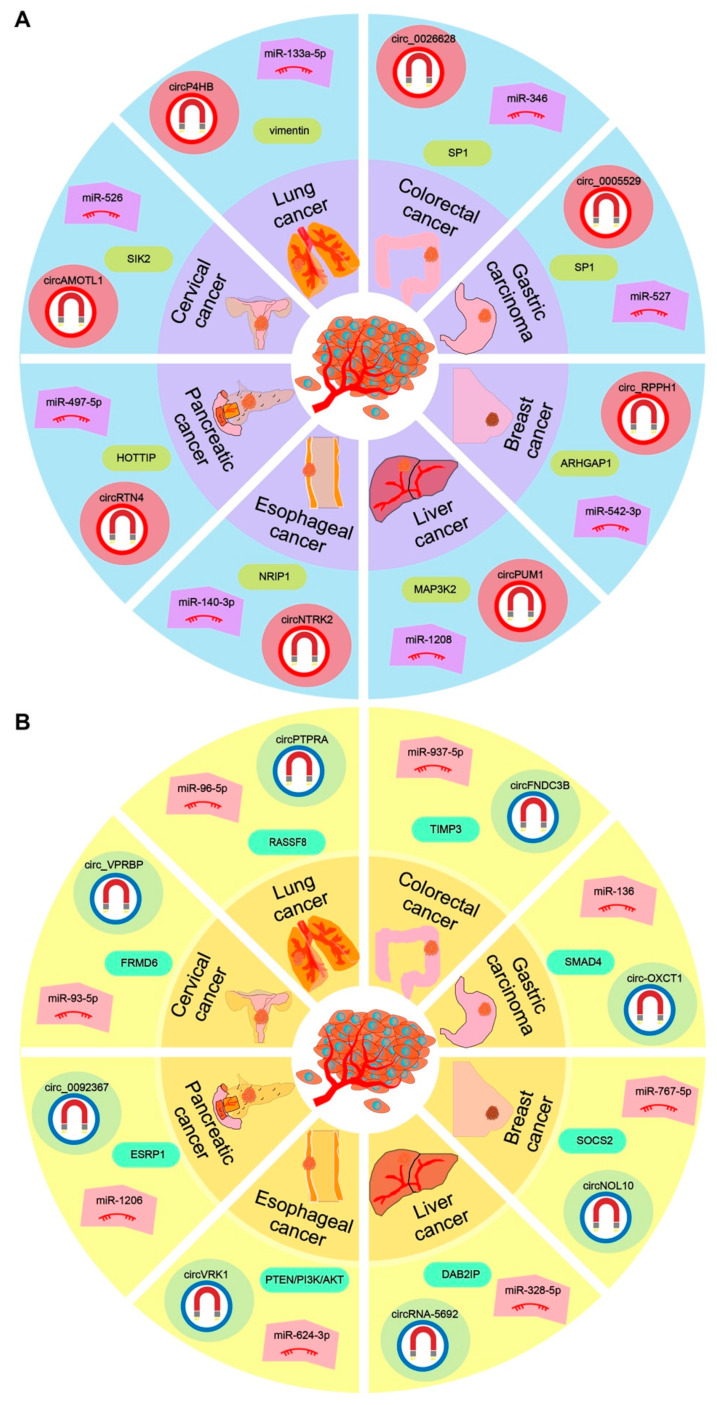
Examples of circRNA-associated ceRNA network based on expression patterns of circRNAs in cancers. (**A**) The RNA interactions based on down-regulated circRNA in specific cancer that may have a potential tumor suppressor role; (**B**) The RNA interactions based on up-regulated circRNA in specific cancer that may have a potential tumor oncogene role.

EMT pathway is related to multiple tumor functions, mainly including tumor initiation, malignant progression, tumor dryness, tumor cell migration, vascular infiltration, metastasis, and resistance to treatment [27,28]. During the progress of EMT, the expression of mesenchymal markers, such as N-cadherin, vimentin, and dense protein, are increased, while the expression of epithelial markers, such as E-cadherin, is decreased. A large number of studies have shown that circRNAs can regulate EMT transcription factors (EMT-TFs: Snail, Slug, Twist, and Zeb family) and EMT-related signaling pathways (TGF-β/Smad, Wnt/β-catenin, PI3K/AKT, MEK/ERK, JAK2/STAT5 and Hedgehog signaling pathways) [29]. Herein, according to the ranking of cancer DALYs (disability-adjusted life years) from 2010 to 2019 [30], we will mainly introduce the detailed molecular mechanism of circRNA regulating the EMT pathway via sponging miRNA in the first eight solid tumors (Figure 2).

### 2.1. CircRNA-Associated ceRNA Network in Lung Cancer

About 85% of lung cancers are non-small cell lung cancer (NSCLC) [31], and many circRNAs have been found with a role in the occurrence and development of NSCLC. Specifically, circP4HB enhances EMT and metastatic disease through miR-133a-5p sequestration, leading to upregulation of vimentin, advocating targeting the circP4HB/miR-133a-5p/vimentin axis as a potential therapeutic option [32], circPTPRA upregulates tumor suppressor RASSF8 by sponging miR-96-5p, and further inhibits EMT and metastasis of NSCLC cell line [33]. Circ_0067934 can act as a competing endogenous RNA to facilitate NSCLC progression by regulating the miR-1182/KLF8 axis and activating Wnt/beta-catenin pathway [34], circ_0000567/miR-421/TMEM100 axis can promote the migration and invasion of lung adenocarcinoma and is associated with cancer prognosis [35], and circ_0020123 interacts with miR-1283 as a ceRNA to regulate PDZD8 expression, thus promoting the proliferation and migration of cancer cells [36]. CircRNA SOD2 upregulates CAMSAP2 via competitively binding miR-2355-5p, and then promotes the progress of NSCLC through the EMT pathway [37]. Circ-0043265 inhibits proliferation, metastasis, and EMT and promotes apoptosis through miR-25-3p/FOXP2 pathway [38]. In addition, circAGFG1 upregulates the expression of ZNF281 via sponging miR-203 to promote EMT and metastasis [39], and circPTCH1 can inhibit the migration and invasion and EMT of lung adenocarcinoma by regulating miR-34c-5p targeting MYCN [40].

### 2.2. CircRNA-Associated ceRNA Network in Colorectal Cancer

Colorectal cancer (CRC) is one of the most common cancers in the world, with 1 to 2 million new cases diagnosed every year, and many circRNAs are associated with CRC. For example, circ1662 binds to YAP1 and accelerates its nuclear accumulation so as to regulate the SMAD3 pathway, which can regulate EMT to promote tumor metastasis [41]; circFNDC3B encodes circFNDC3B-218aa protein to inhibit the expression of snail and then promotes the expression of FBP1 to inhibit the proliferation, migration, and EMT of colorectal cancer [42], and circFNDC3B sequestrates miR-937-5p to derepress TIMP3 and inhibit colorectal cancer progression, a tumor-suppressing role for the circFNDC3B-miR-97-5p-TIMP3 pathway can be detected in CRC, indicating circFNDC3B-enriched exosomes can inhibit angiogenesis and cancer progression [43]; circPTK2 (circ-0005273) promotes the expression of EMT-related proteins by binding to vimentin sites Ser38, Ser55 and Ser82 [44]; circRNA-101951 activates the EMT pathway mediated by KIF3A [45]. CircRNA also contributes to the occurrence and development of CRC via acting as a sponge miRNA. CircRNA-102209 upregulates the RIN1 signal via sponging miR-761 [46], circ-0082182 can down-regulate the expression of miR-411 or miR-1205 [47] and circ_0026628 can activate Wnt/β-catenin pathway by enhancing the interaction between SP1 and β-catenin via sponging miR-346 [48], thus promote the proliferation, invasion, and EMT of CRC cells. Hsa_circ_0006732 regulates colorectal cancer cell proliferation, invasion, and EMT by miR-127-5p/RAB3D axis [49], circ_0085315 can promote cell proliferation, invasion, and migration in colon cancer through miR-1200/MAP3K1 signaling pathway [50], hsa_circ_0007843 can act as a miR-518c-5p sponge to regulate the migration and invasion of colon cancer SW480 cells [51], and circular RNA hsa_circ_0008285 inhibits cell proliferation and migration via the miR-382-5p/PTEN axis.

### 2.3. CircRNA-Associated ceRNA Network in Gastric Carcinoma

Gastric carcinoma (GC) is the fourth most common cancer and the second most common cause of cancer death, and many circRNAs have been studied with a regulatory role in GC. CircNRIP1 can activate AKT1/mTOR pathway by sponging miRNA-149-5p [52], circRNA-100290 up-regulates EMT-related oncogene ITGA11 by sponging miR-29b-3p [53], circ-0032821 can regulate miR-1236-3p/HMGB1 axis [54], and circ_0005529 up-regulates SP1 by sponging miR-527 [55], thus promoting the proliferation, EMT, migration and invasion of GC cells. Circ-OXCT1 suppresses EMT and metastasis by attenuating the TGF-beta pathway through the circ-OXCT1/miR-136/SMAD4 axis [56], circRNA-0005075 suppresses carcinogenesis via regulating miR-431/p53/EMT [57], circ_0006089 promotes gastric cancer growth, metastasis, glycolysis, and angiogenesis by regulating miR-361-3p/TGFB1 [58], hsa_circ_0007967 promotes gastric cancer proliferation through the miR-411-5p/MAML3 axis [59], the circular RNA, circ_0006089, also regulates the IGF1R expression by targeting miR-143-3p to promote gastric cancer proliferation, migration, and invasion [60], the overexpression of circ_0051620 is associated with progress and poor prognosis of GC, promoting the development and metastasis via sponging miR-338-3p and decoying ADAM17 [61], and circFGD4 can inactivate β -catenin signal through miR-532-3p/APC pathway to inhibit the activity, migration, and EMT of GC cells [62].

### 2.4. CircRNA-Associated ceRNA Network in Breast Cancer

Breast cancer (BC) is the fifth leading cause of overall cancer death [63]. There are many circRNAs that can regulate EMT and metastasis and further contribute to the carcinogenic process, and the circular RNA-associated ceRNA network involved in biological processes may be quite crucial in the occurrence and development of cancer. For example, circ_0047303 may be a potential key regulator to mediate the upregulation of key angiogenesis-related genes, including HIF-1, EIF4E2, and VEGFA in TNBC, via sponging the tumor-suppressive miRNAs [64], circ_0000518 promotes breast cancer progression through the miRNA-1225-3p/SRY-box transcription factor 4 pathway, and it is oncogenic in BC [65], circ_0089153 is an oncogene in breast cancer, which enhances proliferation and metastasis via sponging miR-2467-3p/E2F6 [66], circ_IRAK3 exerts a promoting effect on BC progression by modulating the miR-603/KIF2A axis [67], circRNA_000554 up-regulates ZFP36 by sponging miR-182 [68], circ_RPPH1 up-regulates ARHGAP1 via sponging miR-542-3p [69], circ_ZFR up-regulates FABP7 by sponging miR-223-3p [70]. Hsa-circ-0061825 (circ-TFF1) contributes to BC via targeting miR-326/TFF1 [71], and circNOT2 can prevent breast cancer invasion, migration and EMT by regulating twist family BHLH transcription factor via targeting miR-409-3p/Twist1 [72]. CircNOL10, however, can up-regulate SOCS2 and inhibit the JAK2/STAT5 pathway by sponging miR-767-5p, inhibit BC cell proliferation, migration, invasion, and EMT, and slow down the growth of xenograft tumors in vivo [73].

### 2.5. CircRNA-Associated ceRNA Network in Liver Cancer

Primary liver cancer is the seventh most common cancer in the world and the second most common cancer cause of death. Hepatocellular carcinoma (HCC) is the cause of diagnosis and death of most liver cancers, and it is the main type of liver cancer, accounting for about 75% of the total [74]. Similarly, some circRNAs have been concerned because of their roles in liver cancer via sponging flexible regulators, miRNAs. Specifically, knockdown of circ-CSPP1 can suppress HCC development both in vitro and in vivo by upregulation of miR-493-5p and downregulation of HMGB1 [75], circ-CCND1 plays a cancer-promoting role by modulating the miR-497-5p/HMGA2 axis [76], circPUM1 could promote the development of HCC by up-regulating the expression of MAP3K2 via sponging miR-1208 [77], circRNA-5692 can sponge miR-328-5p to enhance DAB2IP expression, and the circRNA-5692-miR-328-5p-DAB2IP regulatory pathway inhibits the progression of HCC [78], circ_CDR1as up-regulates RAF1 expression via targeting miR-1287 and further regulates EMT pathway through MEK/ERK [79], circ_0091579 up-regulates the expression of TRIM27 by acting as a molecular sponge of miR-136-5p [80], and hsa_circ_0003288 enhances the expression of PD-L1 by acting as a sponge of miR-145 to promote EMT [81], migration and invasion of HCC cells, circ_0008194 acts as a sponge of miR-190a to promote AHNAK expression [82], and all of these promote EMT process and HCC proliferation and migration. In addition, circHPS5 upregulates the expression of HMGA2 by acting as a miR-370 sponge, thus accelerating the occurrence and proliferation of HCC tumors and promoting the migration of cancer cells through the EMT pathway.

### 2.6. CircRNA-Associated ceRNA Network in Esophageal Cancer

Esophageal cancer (EC) is listed as the seventh most common cancer in the world, and it is also the sixth leading cause of malignant tumor-related death [83]. In relevant studies about esophageal cancer, hsa_circ_0001741 promotes esophageal squamous cell carcinoma stemness, invasion, and migration by sponging miR-491-5p to upregulate notch3 expression [84], hsa_circ_0023984 can regulate cell proliferation, migration, and invasion via regulating miR-1294/PI3K/Akt/c-Myc pathway [85], ZEB1-mediated downregulation of circ-DOCK5 facilitates metastasis by forming a positive feedback loop with TGF-beta by altering the miR-627-3p/TGFB2 signaling [86], circ_0004771 up-regulates CDC25A by acting as a sponge of miR-339-5p [87], circ_0004370 regulates the miR-1301-3p/COL1A1 axis, and circNTRK2 acts as a sponge of miR-140-3p to up-regulate NRIP1 [87], circ_0006948 acts as miR-4262 sponge to up-regulate FNDC3B [87], and circ-0006948 binds to miR-490-3p and targets the oncogene HMGA2 to induce EMT pathway [88], thus promoting the proliferation, migration, and invasion of EC cells. CircFAM120B is a promising biomarker of esophageal squamous cell carcinoma, which acts as a tumor suppressor via the circFAM120B/miR-661/PPM1L axis and PKR/p38 MAPK/EMT pathway [89]. Further, circVRK1 upregulates PTEN by acting as a molecular sponge of miR-624-3p, decreasing the activity of PI3K/AKT signaling pathway, and further inhibits the proliferation, migration, and EMT of EC cells and reversing the radio-resistance [90], and downregulation of circ_0001273 can inhibit the growth, migration and glutamine metabolism of esophageal cancer cells via targeting the miR-622/SLC1A5 signaling axis, indicating high expression of circ_0001273 contributes to EC progression via modulating the miR-622/SLC1A5 signaling axis [91].

### 2.7. CircRNA-Associated ceRNA Network in Pancreatic Cancer

Pancreatic cancer (PC) is a fatal disease and one of the most aggressive and fatal malignant tumors. Pancreatic ductal adenocarcinoma (PDAC) has been the seventh leading cause of cancer death worldwide [92]. The researchers found that circ-0001666 acts as a sponge of miR-1251, the circRNA-associated ceRNA network further up-regulates SOX4 [93], and circRTN4 regulates the expression of Slug, Snai1, Twist, Zeb1, and N-cadherin in the EMT pathway by interacting with tumor suppressor miR-497-5p [94]. Circ-000510 acts as a sponge of miR-20a-3p and indirectly regulates the expression of COL11A1 [95]; circ-0092314 can promote the proliferation, migration, and invasion of pancreatic cancer cells by binding to miR-671 and alleviating the inhibition of its downstream target S100P [96]; circNEIL3 regulates the expression of ADAR1 by acting as a sponge of miR-432-5p, so as to induce RNA editing of GLI1, and ultimately affect the cell cycle progress and promote EMT [97]. Circ-0013587 increases the level of E-cadherin by decreasing the expression of miR-1227, thus reversing the erlotinib resistance in pancreatic cancer cells and inhibiting the proliferation and EMT of pancreatic cancer cells [98]. In addition, circ-0092367 significantly down-regulates in PC tissues and cell lines by inhibiting the level of miR-1206, up-regulating the expression of ESRP1, finally inhibiting EMT and enhancing the sensitivity of cancer cells to gemcitabine treatment [99].

### 2.8. CircRNA-Associated ceRNA Network in Cervical Cancer

Cervical cancer (CC) is the third most common cancer among women in the world. There are many circRNAs that regulate the metastasis of cervical cancer cells through EMT. CircAMOTL1 upregulates SIK2 by acting as a sponge of miR-526b, promotes AKT signal transduction, and induces EMT [100], circRNA-PVT1 can target EMT of cervical cancer cells induced by miR-1286 through exosomes [101], circ-HIPK3 acts as a sponge of miR-338-3p, regulates EMT mediated by HIF-1α [102], circ-ABCB10 up-regulates ZEB1 by acting as a miR-128-3p sponge [103], hsa_circ_0000730 can restrain cell proliferation, migration, and invasion in cervical cancer via miR-942-5p/PTEN axis, and it probably acts as a tumor suppressor and may be a candidate target for the treatment of CC [104]. Circ-0003221 can inhibit the growth and metastasis of cervical cancer cells via miR-139-3p/S100A14 pathway [105], and circ0036602 is a tumor-promoting circRNA that can promote CC cells by sponging miR-34-5p and miR-431-5p to further regulate HMGB1, and the circular RNA, Circ0036602, may have huge prospects as a potential therapeutic target [106]. Moreover, circ-ACACA may promote CC tumorigenesis and glycolysis by targeting the miR-582-5p/ERO1A signaling axis, indicating circ-ACACA can promote proliferation, invasion, migration, and glycolysis of cervical cancer cells [107]. Circ_VPRBP can inhibit cell proliferation, migration, and invasion and promote cell apoptosis of cervical cancer cells by regulating the miR-93-5p/FRMD6 axis [108].

All of these results strongly support the critical role of circRNA in the carcinogenic process, especially via acting as a miRNA sponge to regulate relevant target mRNAs and further biological processes. Indeed, some circRNAs have been concerned because of their potential clinical application in human cancers. Circ_CDR1as has regulatory effects on multiple axes, such as miR-1270/AFP, miR-1287/Raf1, miR-7-5p/KLF4, miR-641/HOXA9, miR-219a-5p/SOX5, miR-7/HOXB13, and miR-876-5p/MAGE-A, indicating its role in neoplasms and application as a biomarker [109]. More circRNAs have been reported with critical roles in carcinogenesis, especially through acting as miRNA sponges, which have been an important approach to contribute to subsequent relevant biological processes. More importantly, some circRNAs may simultaneously bind to multiple miRNAs, and the interactions between circRNAs and miRNAs are more complex than we thought. The circRNA-miRNA-mRNA ceRNA network will enrich our understanding of cross-talks among different RNAs, and the circRNA-associated ceRNA network may have potential clinical application in precision medicine.

## 3. Potential Roles of circRNAs in Tumor Microenvironment through Interacting miRNAs

After the EMT process, tumor cells may gain the ability to migrate and invade. By interacting with the components in the tumor microenvironment, tumor cells can escape from the immune system, promote survival and metastasis, enter microcirculation, and have distant metastasis, thus producing secondary tumors in other tissues or organs. More evidences show that there is a complex interaction between circRNAs and TME, which plays an important role in regulating TME.

CircRNAs participate in these immunosuppressive networks by impairing normal immune cell function and making tumors escape [110]. For example, circSnx5 can regulate dendritic cells activation and function through the miR-544/SOCS1 axis and inhibit nuclear translocation of PU.1 [111], circ-0007456 is significantly down-regulated in tumor tissues, and circ-0007456 regulates the expression of ICAM-1 in HCC by sponging miR-6852-3p, thus regulating the susceptibility of HCC tumor cells to NK cells [112]. Circ-CPA4 regulates cell growth, dryness, drug resistance, and immune escape of NSCLC via the let-7 miRNA/PD-L1 axis [93]. In Cryptococcal meningitis (CM), circ-0001806 acts as a sponge of miRNA-126, positively regulates the level of Adrenomedullin (ADM), and reduces apoptosis and G1/S arrest in T cells [113]. Hsa-circ-0110102, as a cancer-causing circRNA, inhibits the expression of CCL2 in HCC cells through the sponge effect of miR-580-5p, CCL2 further activates the COX-2/PGE2 pathway in macrophages in a FOXO1-dependent manner, and promotes the progress of hepatocellular carcinoma [114].

Non-cellular components include cytokines, growth factors, extracellular matrix (ECM), and metabolites. It is found that circ-0005519 is abnormally expressed in CD4^+^ T cells of asthma patients, and it can regulate let-7a-5p in CD4^+^ T cells to induce the expression of cytokines IL-13 and IL-6 [115]. CXCL11, a cytokine derived from CAFs, upregulates the expression of circUBAP2, which weakens the inhibition of IFIT1/3 and promotes the migration and metastasis of HCC cells by sponging miR-4756 [116]. NF-κB can mediate cell stress response, cytokine production, and immune response. Additionally, circIKBKB can activate the NF-κB pathway and promote bone metastasis of breast cancer by promoting IKKβ-mediated IκBα phosphorylation, inhibiting the IκBα feedback loop and promoting NF-κB to promote various bone remodeling factors [117]. ECM, including collagen, fibronectin, laminin, glycosaminoglycan, and proteoglycan, is an important tissue barrier for tumor invasion and metastasis. Matrix metalloproteinases (MMPs) play an important role in the pathological destruction of ECM, in the regulation of the tumor microenvironment and in the metastasis of cancer cells. CircRNA-CER plays a “sponge” role by competitively binding to miR-136, regulating the expression of MMP-13, and participating in the ECM degradation process of chondrocytes [118]. Exosomes are extracellular vesicles, usually 30 to 100 nm in diameter, which are secreted by different types of cells in TME and participate in diverse biological processes. Tumor cells can secrete 10 times more exosomes than other cells [119]. In the serum and urine exosomes of bladder cancer, circPRMT5 sponges miR-30c through the snail1/E-cadherin pathway to enhance the EMT process, while circ-PDE8A, from hepatic metastatic PDAC cells, acts as a sponge of miR-338 to up-regulate MACC1 and then increases the invasive growth of PDAC cells, thus affecting the tumor progression [120]. Therefore, circRNAs play potentially important roles in the tumor microenvironment through interacting miRNAs, and a circRNA-associated ceRNA network may be a suitable approach to screening a novel biomarker for cancer.

## 4. Widespread circRNA-Associated ceRNAs and Complexity of RNA Cross-Talks

**Figure 3:**
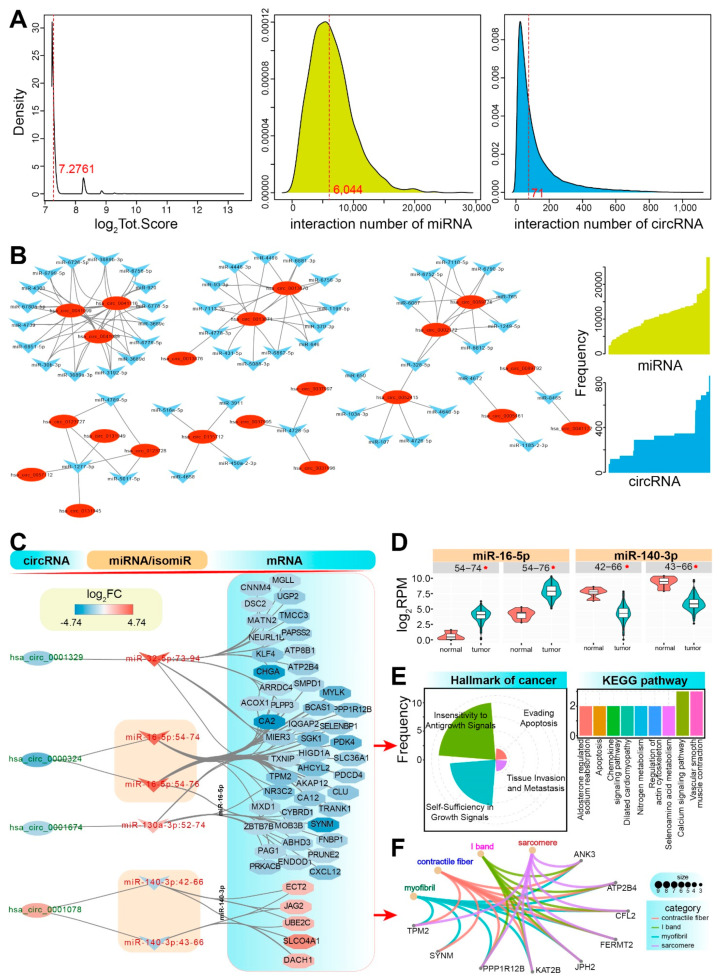
Widespread circRNA-associated ceRNAs and complex RNA interactions among different RNAs. (**A**) The distribution of scores of predicted candidate interactions between miRNAs and circRNAs is presented (data are obtained from Liu et al. [121]), and the median value of log_2_Score is 7.2761; the distribution of interaction number of miRNA, and the median interaction number is 6044; the distribution of interaction number of circRNA, and the median interaction number is 71. (**B**) An example of miRNA:circRNA interaction network shows the potential interactions between the two ncRNAs (all of them are detected with the higher scores). The real interaction number of miRNAs and circRNAs is also presented on the right. (**C**) An example of a potential circRNA-associated ceRNA network shows complex interactions among different RNAs in colorectal cancer. The dysregulated circRNAs are identified using GSE147597 data from the GEO database [124], and dysregulated relevant miRNAs/isomiRs (dominantly expressed) and some of their target mRNAs (dominantly expressed) are further obtained from TCGA-COAD. The miRNA:mRNA interactions are obtained from the starBase database [125,126], and all of these interactions are predicted by at least 3 algorithms. The detailed multiple isomiRs are presented in the network according to the detailed start and end positions on the chromosome. miR-32-5p:73-94 indicates the detailed location of hg38:chr9:109046273-109046294:− of isomiR in miR-32-5p locus; miR-16-5p:54-74 indicates hg38:chr3:160404754-160404774:+ (mir-16-2 precursor) of isomiR in miR-16-5p locus; miR-130a-3p:52-74 indicates hg38:chr11:57641252-57641274:+ of isomiR in miR-130a-3p locus; miR-140-3p:42-66 indicates hg38:chr16:69933142-69933166:+ of isomiR in miR-140-3p locus. These candidate interactions only present the biological relationships and dysregulated expression patterns as examples, and expression correlations are not estimated due to limited data; (**D**) The detailed expression distributions of isomiRs in miR-16-5p and miR-140-3p loci between tumor and normal samples. All of them are significantly up-regulated or down-regulated in tumor samples. * indicates log_2_FC > 1.5 and padj < 0.05 or log_2_FC < −1.5 and padj < 0.05; (**E**) According to involved target mRNAs in Figure 3C, primary functional analysis is performed, mainly including the potential contributions in hallmarks of cancer and KEGG pathways; (**F**) Functional enrichment analysis is performed using clusterProfiler [127].

Based on the potential binding events of circRNA and miRNA, candidate interactions are very common in the ncRNA world [121] (Figure 3A). Similar to miRNA:mRNA interactions, theoretically, many miRNAs can interact with multiple circRNAs, indicating the potential flexible regulatory roles of the small RNAs in the coding-non-coding RNA regulatory network. The interaction network of miRNA:circRNA indicates the complex interactions (Figure 3B), which implicates that some miRNAs can be interacted with by many circRNAs that may further perturb target mRNA expression. More importantly, in the miRNA locus, the phenomenon of multiple isomiRs further complicates the ceRNA regulatory network [122] (Figure 3C), because these isomiRs are always involved in diverse sequence and expression patterns (Figure 1C,D). The canonical miRNA sequence may not be the most dominantly expressed isomiR sequence, while many isomiRs are unexpectedly dominantly expressed, which ensures further regulatory roles in the regulatory network. Some isomiRs are involved in changed 5′ ends and seed sequences, which will complicate and potentially perturb coding-non-coding RNA interaction networks due to gaining and/or losing mRNA targets caused by changed seeds [15,123]. Herein, in the miR-140-3p locus, 2 isomiRs are primarily screened as relevant miRNA sequences, but the two isomiRs are involved in 1 nucleotide divergence at the 5′ end (Figure 3C). Despite no further target mRNAs being specifically precited based on the detailed isomiRs, the changed seed sequence actually can lead to a greater gain and/or loss of targets than its canonical miRNA, which results in a complex and rewired regulatory network. Indeed, the expression patterns of diverse isomiRs may diverge or be similar despite the fact that they are yielded from the same miRNA locus, but similar dysregulated patterns can be found, even 5′ isomiRs with the shifted seeds (novel seeds) are also detected abundant expression patterns (Figure 3D). These expression patterns implicate strict regulatory patterns in miRNA mature processes, which contribute to the stable regulatory roles in multiple biological processes. The isomiR landscapes provide potential multiple selection and competition for circRNA, which complicates the circRNA-miRNA/isomiR-mRNA interaction or RNA.

The binding events of circRNA and miRNA/isomiR will cause abnormal expression of target mRNA, which may further perturb relevant biological processes and pathways. If the interactions are comprehensively estimated at the isomiR level, more complex interactions can be found that would rewire the RNA interaction network. Based on the role of circRNA as a miRNA/isomiR sponge, relevant target mRNAs may be influenced, especially for those mRNAs with critical roles in multiple biological processes. For example, some target mRNAs contribute to hallmarks of cancer, Kyoto encyclopedia of genes and genomes (KEGG) pathways and Gene Ontology (GO) terms (Figure 3E,F), especially insensitivity to antigrowth signals and self-sufficiency in growth signals, calcium signaling pathway and vascular smooth muscle contraction, etc. These potential contributions to multiple important biological processes implicate that the dysregulation expression of these target mRNAs caused by abnormal miRNA expression due to the role of sponge competition of circRNA may be a critical approach in the occurrence and development of cancer. Taken together, a series of RNA interactions or cross-talks will further perturb subsequent biological processes that may be critical in the human carcinogenic process, implicating the corresponding roles of circRNAs as potential diagnostic biomarkers and therapeutic targets in future cancer treatment.

## 5. Challenges and Future Perspectives

The poor survival rate, high recurrence rate, and drug resistance of metastatic cancer patients are the main obstacles in current clinical treatment. An interesting way to improve cancer treatment is to use cancer-specific expression profiles to develop biomarkers for the progression from primary diseases to metastatic diseases [128]. A qualified biomarker should have good sensitivity, specificity, repeatability, stability, and clinical utility [129]. The specificity of RNA expression patterns, such as in exosomes, saliva, plasma, and blood, provides an ideal and potential candidate biomarker, which can be used for cancer treatment and diagnosis and further promotes precision medicine.

CircRNAs may have potential application in the cancer progression, diagnosis, prognosis, treatment, and drug resistance, and may be a potential therapeutic target. The most likely and common treatment for overexpressed circRNA is to use siRNA targeting its sequence [130]. Similar to the method in vitro, the designed siRNA should aim to the unique reverse splicing point of cancer-causing circRNA to avoid any interference with linear host genes. Recently, a cre-lox system has been used to knock out or knock down circRNA in specific cells in vivo [131]. CRISPR/Cas9 has been used to destroy the intron pairing on both sides of the circular exon in the biological process of circRNA to knock out circRNA. CRISPR/Cas13 system specifically targets single-stranded RNA and directly targets the back splicing connection of circRNA to knock down circRNA [132]. For the down-regulated circRNA, the level of tumor suppressor gene circRNA can be restored by cloning the circRNA sequence and its regulatory flanking region [133], or the over-expression of circRNA can be realized by artificially synthesizing circRNA [134].

The main problem of circRNA as a therapeutic method is to find a vector to deliver siRNA or express circRNA in vivo [135], and nanoparticles have been used to realize the delivery of circRNA. During delivery, they can protect RNAi molecules from degradation, promote cell uptake and prevent immune activation, and can also be used to deliver circRNA expression plasmids. Nanoparticles may be a promising delivery system for circRNA targeting agents [136]. Another method is to transmit circRNA through exosomes [131]. Similar to the nanoparticle delivery system, exosomes can protect RNAi molecules from degradation, promote cellular uptake and not trigger an immune response, while being much safer than synthetic nanoparticles. However, it is difficult to produce exosomes containing circRNA for a specific therapeutic purpose, which is a future challenge for precision medicine.

In addition, based on the important roles of circRNA-associated ceRNA network in cancer, more studies should be performed to understand the detailed interactions and potential molecular mechanisms between circRNA and miRNA/isomiRs, especially for those isomiRs with novel 5′ ends and functional seed sequences. Most importantly, it is an open question whether these multiple isomiRs with diverse sequence and expression and heterogeneities have a potentially competitive relationship during interaction with circRNAs, and even lncRNAs that are also a class of major miRNA sponges. Similar interaction patterns also exist in homologous miRNA loci, and the potential cross-talks among diverse RNAs and subsequence biological processes may contribute to systematically exploiting the interesting ncRNA world.

## 6. Conclusions

Based on the important roles of circRNAs in tumorigenesis, particularly in tumor metastasis via acting as sponges of miRNAs, we review the types, characteristics, and biological functions of circRNAs. CircRNAs play multiple roles in tumor metastasis via sponging miRNAs, which can promote angiogenesis, enhance the migration and invasion ability of cancer cells through EMT, and interact with TME to promote cancer metastasis, thus regulating tumor progression. Further, the circRNA-associated ceRNA network is more complex than we thought, especially for the phenomenon of multiple isomiRs in the miRNA locus, which will greatly enrich our understanding of the ncRNA world and their contributions to the human carcinogenic process and potential clinical applications in cancer treatment.

Taken together, it is essential to understand the detailed molecular mechanisms underlying the multiple roles of circRNAs in cancer metastasis via sponging miRNAs/isomiRs, as this may lead to the development of novel diagnostic tools and treatment targets for cancers.

## Data Availability

Not applicable.
